# Supplementary material for: Conditional chemoconnectomics (cCCTomics) as a strategy for efficient and conditional targeting of chemical transmission
Source: eLife. 2024 Apr 30;12:RP91927. doi: 10.7554/eLife.91927 (PMC11060718; doi:10.7554/eLife.91927)
Supplement: Figure 3—source data 3. [file elife-91927-fig3-data3.zip › Figure 3 source data3 part 1.zip!/Figure 3-Source data 3-part1/1071f3-1-CRISPResso_Report_jvi77nmx.zip!/CRISPResso_on_1071f3_2/4d.Position_dependent_average_indel_size.pdf]

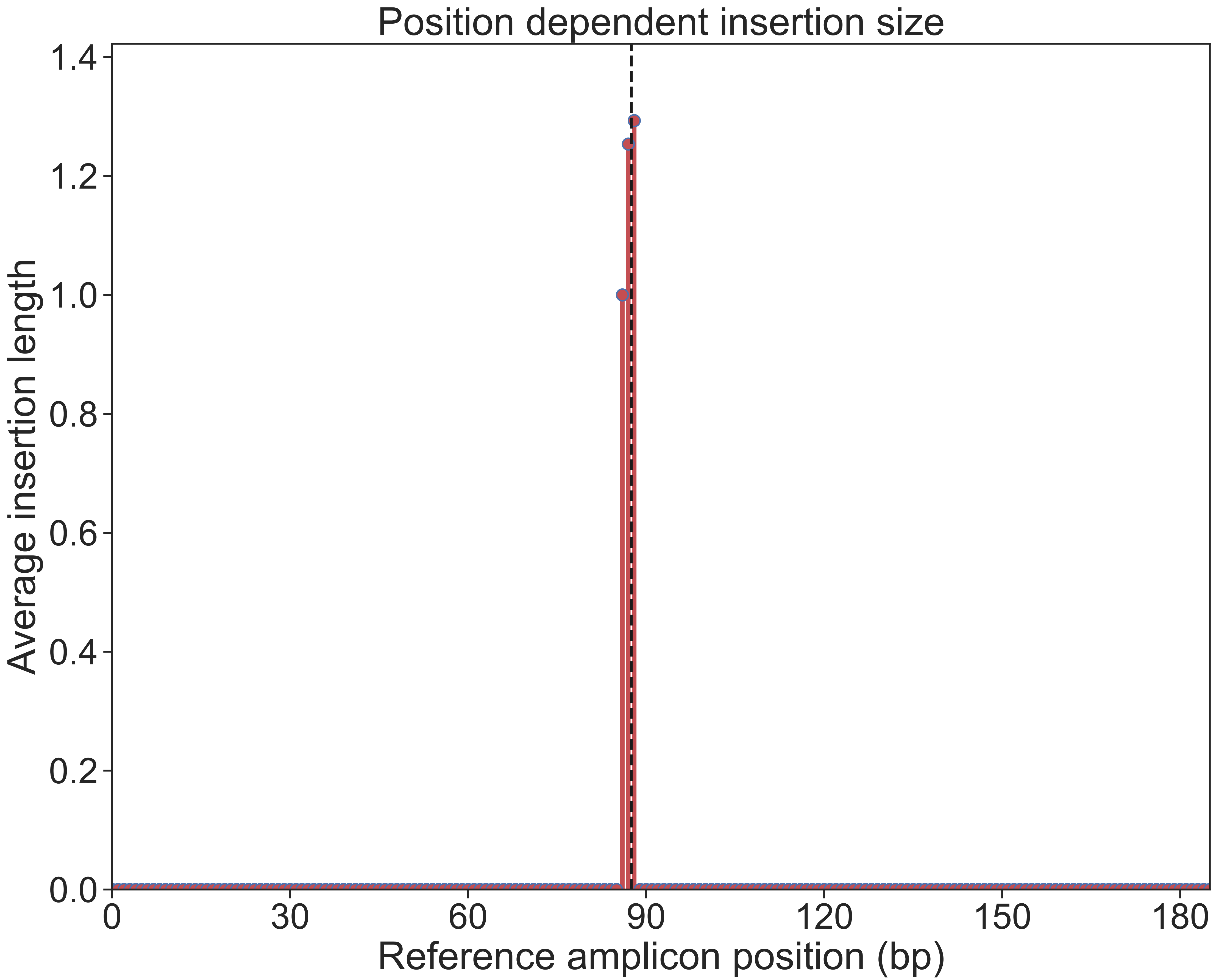

Position dependent insertion size
Average insertion length
1.4
1.2
1.0
0.8
0.6
0.4
0.2
0.0
0
30
60
90
120
150
180
Reference amplicon position (bp)

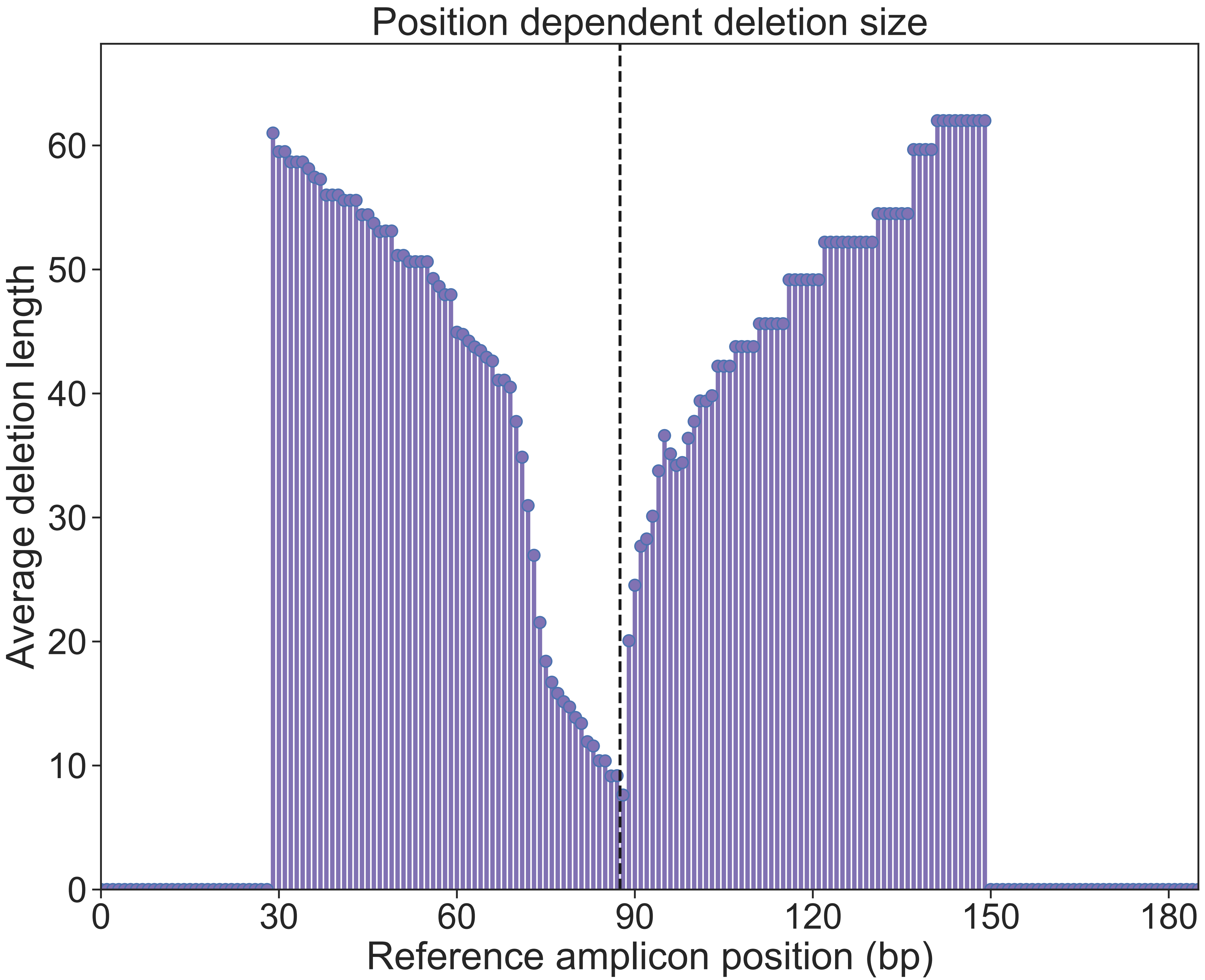

Position dependent deletion size
Average deletion length
60
50
40
30
20
10
0
0
30
60
90
120
150
180
Reference amplicon position (bp)
